# Acromioclavicular joint dislocation treated with Bosworth screw and additional K-wiring: results after 7.8 years – still an adequate procedure?

**DOI:** 10.1186/s12891-017-1692-0

**Published:** 2017-08-04

**Authors:** Thomas M. Tiefenboeck, Domenik Popp, Sandra Boesmueller, Stephan Payr, Julian Joestl, Micha Komjati, Harald Binder, Mark Schurz, Roman C. Ostermann

**Affiliations:** 1 Department of Trauma Surgery, Medical University of Vienna, Waehringer Guertel 18-20, A-1090 Vienna, Austria; 2 AUVA Trauma Center Meidling, Vienna, Austria; 3 Department of Orthopaedics, Hospital of sacred Heart of Jesus, Vienna, Austria

**Keywords:** AC dislocation, Long-term outcome, Bosworth screw, K-wiring

## Abstract

**Background:**

The acromioclavicular (AC) joint dislocation is a major reason for shoulder instability. Different concepts of treatment and surgical methods are described in the literature. Thus, the purpose of this study was to present our data of long-term follow-up of patients having undergone treatment of acromioclavicular (AC) joint dislocation using the Bosworth Screw with additional K-wiring.

**Methods:**

This study was conducted as a retrospective single centre data analysis. All patients treated operatively for AC joint dislocation with a Bosworth screw and additional K-wire fixation at our Department were asked to participate in this study.

**Results:**

The study population consisted of 22 patients, 20 male and 2 female, with a mean age of 40 years ±15.6 years. Three grade-II lesions, 13 grade-III lesions, four grade-IV lesions and two grade-V lesions according to the Rockwood classification were found. The overall mean clinical outcome at the latest follow up was: Constant 95, DASH 6.4, ASES 94.6, SST 99.02, UCLA 33.1, ACJI 91.82 and VAS 0.29 – representing a good-to-excellent long-term outcome in all patients after at least 2 years follow-up (range; 2 - 19 years). Overall, 19 patients (86%) reported to be very satisfied with the achieved result, 15 patients (68%) reported to be able to participate in every sports activity and 16 patients (73%) reported to be able to perform their daily work without limitations. Overall, complications occurred in three patients (14%). Only one patient remained unsatisfied with the achieved result.

**Conclusion:**

Summarizing, our reported results showed that surgical fixation of acute AC joint dislocation with a Bosworth screw and additional K-wire fixation leads to good-to-excellent functional outcome and highly satisfactory results in the majority of patients. Despite its complications, in accordance with our results, Bosworth screw fixation with additional K-wiring in AC joint dislocation represents an adequate surgical procedure.

Level of Evidence: Level IV, retrospective study.

## Background

The acromioclavicular (AC) joint dislocation is a major reason for shoulder instability, typically associated with a direct high-energy trauma to the shoulder or strong force on the outstretched arm [1–3]. It often occurs in young and athletic patients emphasizing the importance of restoring normal anatomy and function for full recovery [4]. Different concepts of treatment and surgical methods, including a variety of implants, are described in the literature [3, 4], however, no gold standard technique has yet been implemented [5]. Rockwood I and II lesions are normally treated non-surgically [6]. Surgical treatment is recommended for grade IV and V lesions according to Rockwood classification; however, there is still a controversy of grade III lesions to present an indication for surgery.

The use of Synthetic materials for fixation has become more popular, but despite a rapid development, these techniques can lead to serious complications such as incomplete reduction, ligament failure, foreign body reaction and fracture of the clavicle [7, 8]. Metal implants offer immediate stabilization but are also often limited by implant failure [3]. The clavicle hook plate is often associated with a symptomatic impingement [9]. Recently, anatomic reconstruction of the CC ligaments with autogenous grafts has become popular. The theory was that the graft enabled natural healing of the torn CC ligaments [10, 11]. However, to this day, only limited data is available regarding this technique [10] even when used in acute AC dislocations [12].

Another surgical method is the coracoclavicular fixation with the Bosworth screw, which has shown to be an effective surgical procedure for treating complete grade-III, −IV and -V AC joint dislocations [13]. Excellent short- to mid-term (2.5 months – 3 years) results using this technique are described in the literature [14, 15]. A biomechanical study revealed that the Bosworth screw restored strength to the AC joint equivalent to the intact native coracoclavicular ligaments [13]. Nevertheless, in all patients a second surgical intervention is needed to remove the implant [16].

However, long-term outcome after AC joint reconstruction by coracoclavicular fixation with the Bosworth screw and additional K-wiring has not yet been presented in the literature.

Therefore, the aim of this study was to present the long-term functional outcome and to evaluate safety and efficacy after AC joint reconstruction with a Bosworth screw and additional K-wiring. We hypothesized that patients who underwent AC joint reconstruction with the Bosworth screw and additional K-wiring presented with a good-to-excellent outcome at a long-term.

## Methods

The approval for this study was granted by the Ethics Committee of the Medical University of Vienna (Borschkegasse 8b/E06, 1090 Vienna, Medical University of Vienna EK. No. 1218/2015) and the study itself was conducted according to the Declaration of Helsinki in its latest amendment.

**Fig. 1:**
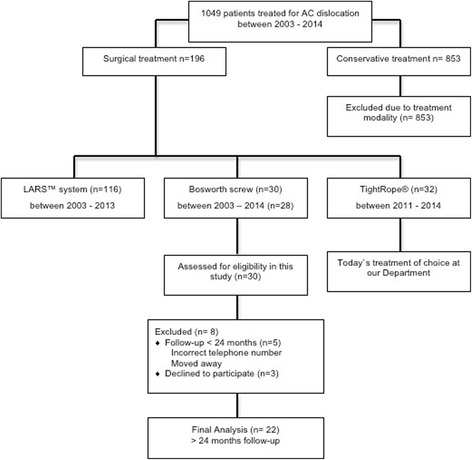
Overview of the different surgical treatment modalities of AC joint dislocation at our Department between 2003 and 2014

Our study was conducted as a retrospective follow-up study at the Department of Trauma Surgery at the Medical University of Vienna. All database files and medical records of patients treated with Bosworth screw fixation and additional K-wiring for an acute AC joint dislocation were retrospectively reviewed for clinical and radiological outcome. Three different surgical methods were used at our Department to treat AC dislocations – Bosworth screw and K-wiring, reconstruction with the LARS™ system and the TightRope® system. In the last years the TightRope® system was used for reconstruction in most cases. Figure 1 offers an overview of surgical AC treatment at our Department.

Inclusion criteria were as follows: (1) patients aged >18 years, (2) follow-up of at least 24 months, (3) AC dislocation treated with Bosworth screw and additional K-wiring and (4) no further concomitant injuries. Excluded from final analysis were: (1) age < 18 years, (2) clinical follow-up less than 24 months, (3) incomplete data set and (4) patients with additional injuries to the shoulder girdle.

In total, 30 patients (3 female / 27 male) with a mean age of 41 years (±15.8 years) had undergone AC joint reconstruction using the Bosworth screw and additional K-wiring between 1995 and 2013 and were recruited for retrospective follow-up after a mean duration of 94 months (± 51.7). Patients were only included if they had a minimum follow-up duration of at least 24 months. This finally left 22 patients with a mean follow-up of 94 months (range: 24 – 233, median: 83). Detailed information on inclusion and exclusion criteria is presented in Fig. 1.

Initial diagnosis was made by a clinical assessment including range of motion (ROM) of the shoulder joint as well as by imaging using x-ray. When patients presented in the acute phase, joint tenderness and horizontal stability was tested, if tolerated by the patient due to the pain. Horizontal stability is tested with the examiner positioned behind the patient. The scapula is fixed with one hand while the other hand tests the horizontal stability by moving the lateral end of the clavicle anteriorly and posteriorly. This was done for both the injured and uninjured side. The peripheral neurovascular status was tested routinely.

Radiographs of the AC joint were performed pre-operatively, post-operatively and at final follow-up in all patients. The radiological examination consisted of standard radiographs (i.e. radiographs according to Rockwood, the “serendipity view”) for both AC joints and axillary radiographs for the injured shoulder. Stress radiographs of both the injured and uninjured AC joint were performed preoperatively and at latest follow-up. Radiographic abnormalities were recorded at pre-, postoperative and latest follow-up when present. An additional CT- or MRI-scan was only performed in a few exceptional cases.

**Fig. 2:**
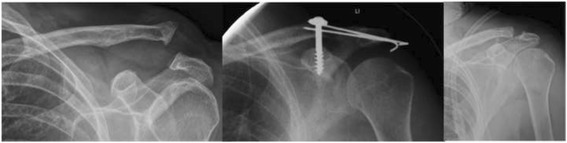
Shows an exemplary case of a Rockwood IV AC dislocation; initial injury X-ray; X-ray after treatment with Bosworth screw and K-wiring; and final result after removing the implants

All surgical procedures were performed by skilled shoulder surgeons after an average duration of 9 days after injury (range 0-24 days), excluding 2 patients who were surgically treated several years after the initial injury due to chronic instability and pain. One of these two patients presented initially with a Rockwood II finally needing surgical treatment because of recurrent pain. The reconstruction of the AC joint was performed according to well-established criteria, which were first described by Bosworth et al. in 1949 [17]. Reconstruction of the AC joint was performed according to well-established criteria, which were first described by Bosworth et al. in 1949 [17]. Patients were positioned in a beach chair manner and - as recommended by the company’s technical guide - surgery was carried out using an image intensifier to confirm correct reduction on the one hand and optimal position of the drill hole on the other. Surgery was performed with a mini-open technique with a sagittal incision of 2 to 3 cm about 3 cm medial of the AC joint. After reduction of the AC joint with a raspatory, two K-wires were introduced parallel and percutaneously from the lateral side through the acromion and the AC joint into the lateral clavicle, thereby achieving a temporary transfixation. In the next step the holes for the Bosworth screw were drilled with a 3.5 mm power drill from the lateral clavicle into the coracoid process. After that a 6.5 mm thread was drilled in the clavicle and the coracoid process. After length measurement a Bosworth screw (DePuy Synthes, Oberdorf, Switzerland) in adequate length was introduced. The K-wires were left in nearly all patients until healing of the ligaments and removed together with the Bosworth screw after a mean duration of 2.4 months (range; 0.6 to 4.8 months) (Fig. 2.)

From our experience, an initial reduction of the AC joint dislocation with K-wires helps with implanting the Bosworth screw in the optimal position without losing the initial reduction, and is therefore the standard procedure at our institution. However, a disadvantage of the additional use of K-wires might be the potential migration or wire breakage. From our experience, an initial reduction of the AC dislocation with K-wires helps with implanting the Bosworth screw in the optimal position without losing the initial reduction, it is therefore the standard procedure at our Department. However, a disadvantage of the additional use of K-wires might be the potential migration or wire breakage.

Postoperatively, shoulders were protected with an arm-pouch sling for 4 weeks. Passive range-of-motion exercises were started on the second post-op day. During the first 4 weeks only pendulum exercises were allowed. After 4 weeks, the patient was allowed to start active mobilization of the shoulder joint upto 90 degrees abduction and flexion up until approximately 8 weeks when the screw should be removed under a short general anaesthesia.

Mobilization is only allowed below shoulder level because above 90 degrees of abduction and flexion, the clavicle undergoes rotation in the coronal plane and hence the screw will certainly loosen but may also break. Sports and heavy weight bearing were not allowed before 12 weeks postoperatively.

All patients included in this study participated in a prospective follow-up assessment, which consisted of a clinical examination including ROM of the shoulder, imaging including x-ray (of the operated and the contralateral side), internationally validated shoulder scores (Constant Score [18], SF 36 Score [19], DASH Score [20], VAS Score [21], ASES [22], UCLA [23], SIMPLE SHOULDER TEST [24], Acromioclavicular Joint Instability Score [25]), assessment of patient satisfaction with the surgical result, sporty activity level and daily work ability (very satisfied – satisfied – not satisfied).

Twenty-two patients (73%) were available for long-term follow-up and signed informed consent prior to participation.

### Statistical analysis

Descriptive data (mean, median, range, proportions) are reported for the entire patient cohort. Differences between means and proportions were tested with the Chi-square test for categorical variables and the unpaired t-test for continuous variables. Additionally, we used repeated ANOVA tests for continuous variables with Bonferroni adjusted post-hoc analysis.

Statistical significance was set with a *p*-value of ≤0.05. Statistical analysis was performed using Microsoft Excel®, SPSS® software (Version 22.0, SPSS Inc., Chicago, IL, USA).

## Results

Of all surgically treated AC joint dislocations at our Department, 18% (*n* = 29) were treated with Bosworth screw and additional K-wiring. 22 of the 30 patients (73%) were available for follow-up after a mean duration of 7.8 years (range: 2-19 years) post injury. None of the patients died during the follow-up period. 20 patients (91%) were male and 2 (9%) were female, with a mean age of 41 years (± 15.8 years, median: 36 years; range: 18 to 76 years).

The mechanisms of injury were as follows: falls in 23% of cases (*n* = 5); sports injuries in 23% (*n* = 5); car accidents in 27% (*n* = 6); and bicycle accidents in 27% (*n* = 6).

In all cases, initial x-rays (lateral, axial, and x-rays under load compared to the healthy side) were performed at the time of first presentation as part of the standard diagnostic procedure for this type of injury at our department.

In three patients an additional MRI was performed, two of these patients presented with chronic instability after 445 and 295 days; all other patients were treated on average after 9 days (range: 0 to 24, mean: 8, SD: 7).

### Classification and treatment

According to Rockwood et al. [26], the following types of AC joint dislocations were found: three grade-II lesions, 13 grade-III lesions, four grade-IV lesions and two grade-V lesions. In all three patients, treated surgical for a grade II lesion, treatment started first conservative, however, due to persisting pain, feeling of instability and patients will they finally got operated.

Based on the time of treatment, 91% (*n* = 20) of the dislocations were classified as acute (< 4 weeks following injury) and 9% (*n* = 2) were classified as chronic (> 4 weeks following injury).

Patients who were not treated immediately after injury were immobilized with a triangular arm sling or a figure-of-eight bandage until surgery. The two patients who were classified as chronic were first treated with a figure-of-eight bandage for 3 weeks and afterwards underwent physiotherapy.

### Clinical outcome

**Fig. 3:**
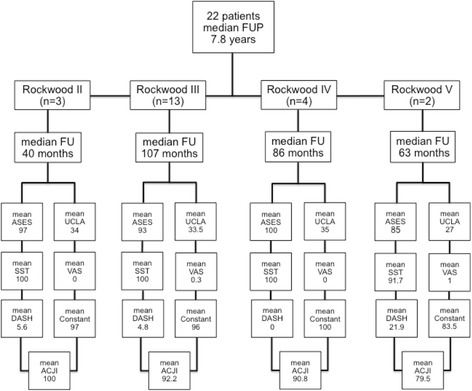
Presenting the results of the surgically treated AC dislocation in regard to the Rockwood classification

The overall mean clinical outcome was: Constant 95, DASH 6.4, ASES 94.6, SST 99.02, UCLA 33.1, ACJI 91.82 and VAS 0.29 – representing a good-to-excellent long-term outcome in all patients at a mean of 7.8 years (Fig. 3).

Table 1 Outlines the outcomes of the SF 36 score.

**Table 1:** Mean SF36 scoring at latest follow-up

Cases	pfi	rolph	rolem	social	mhi	pain	vital	ghp
22	98.06	98.61	90.92	97.92	84.67	92.78	71.11	79.89

*ghp* general health perceptions index; *mhi* mental health index; *pain* bodily pain index; *pfi* physical function index; *rolem* role-emotional index; *rolph* role physical index; *social* social functioning index; *vital* vitality index

Overall, 19 patients (86%) reported to be very satisfied with the achieved result at the latest follow-up. Fifteen patients (68%) reported to be able to do every desired sports activity without limitations. Sixteen patients (73%) reported to be able to perform their daily work without limitations.

Only two patients reported to be highly unsatisfied with the achieved result and reported this in all three questions (satisfaction, sporty activity and daily work). These two patients complained of a painful arc from 120° to 170°. None of the other patients complained of a painful arc at final follow-up and presented with no limitations in their ROM. The remaining patients reported minor limitations regarding work and sports activities.

Patients who experienced complications were less satisfied compared to those without, however, this was not statistically significant (*p* > 0.066).

### Radiological outcome

All patients underwent x-ray imaging of the AC joint under load of the injured and the non-injured side at the latest follow-up. The mean difference in AC and CC joint space between the injured AC joint and healthy AC was significantly reduced after surgery. There was no difference presented in radiographs between post-operative and latest follow-up in regard to AC or CC distance (Table 2).

**Table 2:** Mean AC and CC difference of the injured AC joint compared to the healthy AC joint

Mean CC difference in cm			*p*-value
pre- vs. post-operative	0.83 (0.18 to 1.47)	0.21 (0.01 to 0.86)	*p* < 0.0001
pre-operative vs. latest FUP	0.83 (0.18 to 1.47)	0.22 (0.00 to 0.72)	*p* < 0.0001
post-operative vs. latest FUP	0.21 (0.01 to 0.86)	0.22 (0.00 to 0.72)	*p* = 0.7963
Mean AC difference in cm			
pre- vs. post-operative	0.52 (0.14 to 1.05)	0.19 (0.01 to 0.47)	*p* < 0.0001
pre-operative vs. latest FUP	0.52 (0.14 to 1.05)	0.21 (0.01 to 0.63)	*p* < 0.0001
post-operative vs. latest FUP	0.19 (0.01 to 0.47)	0.21 (0.01 to 0.63)	*p* = 0.7191

*FUP* follow-up; vs. versus

In addition, calcifications of the ligaments were observed in 7 patients (32%). Overall, four patients (18%) presented with signs of early osteoarthritis around the AC joint on the last radiographs.

### Implant removal

All but one patient had the Bosworth screw and the K-wires removed after a mean period of 2.4 months (range, 0.6 to 4.8, median 2.07). This patient returned to hospital after 9 years because of unknown reasons. X-rays showed an intact Bosworth screw - with K-wires still in situ - which were taken out without any complications.

### Complications

**Fig. 4:**
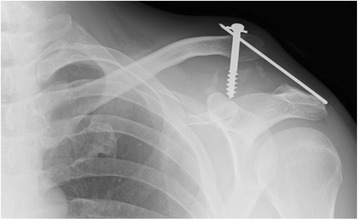
Shows one case of implant failure prior to removal

Overall, complications occurred in three patients (14%). One patient presented with a wound-healing problem, which was treated conservatively with oral Clindamycin 1800 mg until inflammatory markers normalised. In one patient the implanted Bosworth screw and K-wire construct loosened and needed to be removed after 18 days (Fig. 4). The patient was treated conservatively from that time point on. In another patient, the Bosworth screw and the K-wires needed to be removed due to irritation under the skin. All three patients presented free of complications at the latest follow-up. In two of these patients good-to-excellent results could be reached at the most recent follow-up. One patient remained unsatisfied with the end result. In all three patients the initial injury was classified as Rockwood III.

## Discussion

Summarizing, our results present an overall excellent clinical and functional outcome at long-term follow-up in 86% of the patients. In only two patients a painful arc was observed at the final follow-up. The SF 36 score revealed a high patient satisfaction with the achieved result in almost all patients and a high quality of life at long-term follow-up after the surgical procedure, confirming our primary hypothesis. However, in 14% of our patients, complications occurred leading to an unsatisfactory outcome in one patient.

Twenty years ago studies already showed that the Bosworth screw method provided good-to-excellent results in a wide range of 60 to 90% [27, 28]. Nowadays, the rate of good-to-excellent results has risen to up to 90% [16], which was also proven in our study, with a satisfaction rate of 86%. The question today remains whether the Bosworth screw with additional K-wiring is still an adequate procedure for treating AC joint dislocation despite newer surgical procedures like the TightRope® or endobutton fixation are available. These devices, when used correctly, do not require a second operation for implant removal and may therefore be preferred by patients and surgeons. However, a recent study by Darabos N. et al. [16] compared TightRope® vs Bosworth screw fixation showing better results in the TightRope® group, yet not showing any significance in their results. Finally, it is important to consider that the surgical treatment of Rockwood III dislocations remains controversial in the current literature [6].

Overall, our results of functional scores like the Constant Score and patient satisfaction seem to be consistent with the current literature [15, 16]. Furthermore, 68% of our patients were able to do every sports activity and 73% reported to be able to perform their daily work without limitations. Only two patients (9%) showed severe limitations in sports activities and at work.

Major advantages of this technique are its cheapness [16] and the relative simplicity of the surgical technique [29]. The only obvious disadvantage is the necessity of implant removal after approximately 6 weeks, the way it was performed in our patients [30]. Secondary surgery is associated with a potential risk of peri- and post-operative complications. In the present study, surgical complications were documented in three patients (infection, implant dislocation and prominent Bosworth screw and K-wires). There were no complications reported after implant removal in all of our patients. Radiologically, calcifications of the ligaments were seen in 32% and signs of early osteoarthritis in 18% of patients, however, not influencing the clinical and functional outcome. Patients who experienced complications were less satisfied with the result compared to those without.

No signs of implant loosening or failure of the procedure by increasing CC and AC distances could be detected. The mean CC and AC distance of immediate postoperative images compared to images at the latest follow-up did not reveal any statistically significant difference leading to the assumption that the initially reached results could well be maintained.

### Limitations

This study has several limitations, mainly caused by the type of design. As this is a retrospective study, with a small sample size, all patients treated with a Bosworth screw for AC dislocation, regardless of age and sex, were included into this analysis. Due to the small patient number this study is underpowered and therefore drawn conclusions are limited.

Only one method was evaluated without a comparative group. Pre-operative clinical measurements regarding clinical scores are missing. Therefore it was not possible to compare the degree of improvement in the clinical scores. However, with a mean follow-up duration of 7.8 years, this study presents one of the longest mean follow-ups using this surgical technique in the current literature.

## Conclusion

Despite complications, and as our results demonstrate, Bosworth screw fixation in AC joint dislocation represents an adequate and easy to perform surgical procedure, however, there is still an uncertainty between surgical and non surgical treatment. Finally, in contrast to newer techniques, this one always requires a second surgery for final implant removal and this should be kept in mind when using this technique.

## Abbreviations

AC: Acromioclavicular
ASES: American Shoulder and Elbow Surgeons
CC: Coracoclavicular
DASH: Disability of the Arm, Shoulder and Hand Score
SST: Simple shoulder test
UCLA: University of California at Los Angeles Shoulder Score
VAS: Visual analog scale
